# Platelet-rich fibrin as an apical barrier for MTA placement in the treatment of teeth with open apices: a pilot study

**DOI:** 10.1186/s12967-024-05318-0

**Published:** 2024-05-25

**Authors:** Van-Khoa Pham, Tran-Lan-Khue Pham, An-Tran Pham, Hoang-Lan-Anh Le, Thi-Bich-Van Tran, Kim-Khang Huynh, Nguyen-Minh-Hieu Tran, Thuan-Loc Tran, Minh-Hong Tran, Thi-Anh-Thu Tran, Thi-Tam-Duyen Nguyen, Ngoc-Phuc Nguyen

**Affiliations:** 1 University of Medicine and Pharmacy at Ho Chi Minh City, Ho Chi Minh City, Vietnam; 2 National Hospital of Odonto-Stomatology, Ho Chi Minh City, Vietnam; 3 Tra Vinh University, Tra Vinh City, Vietnam; 4 Van Lang University, Ho Chi Minh City, Vietnam

**Keywords:** PRF, MTA, Apical barrier, Open apex, Periapical lesion

## Abstract

**Objective:**

The aim of the present pilot study was to assess the effectiveness of the platelet-rich fibrin (PRF) apical barrier for the placement of MTA for the treatment of teeth with periapical lesions and open apices.

**Methods:**

A total of thirty teeth on twenty-eight patients with open apices and periapical periodontitis were enrolled and divided into two groups in the present pilot study. In the PRF group (fourteen teeth in thirteen patients), nonsurgical endodontic treatment was performed using PRF as an apical matrix, after which the apical plug of the MTA was created. For the non-PRF group (fourteen teeth in fourteen patients), nonsurgical endodontic therapy was performed using only the MTA for an apical plug with no further periapical intervention. Clinical findings and periapical digital radiographs were used for evaluating the healing progress after periodic follow-ups of 1, 3, 6, and 9 months. The horizontal dimension of the periapical lesion was gauged, and the changes in the dimensions were recorded each time. The Friedman test, Dunn-Bonferroni post hoc correction, and Mann-Whitney U test were used for statistical analysis, with *P* < 0.05 serving as the threshold for determining statistical significance.

**Results:**

All patients in both groups in the present pilot study had no clinical symptoms after 1 month, with a significant reduction in the periapical lesion after periodic appointments. The lesion width of the PRF group was significantly smaller than that of the non-PRF group in the sixth and ninth month after treatment.

**Conclusions:**

PRF is a promising apical barrier matrix when combined with MTA for the treatment of teeth with open apices and periapical periodontitis. Small number of study subjects and the short time of follow-up period limit the generalizability of these results.

**Trial registration:**

TCTR, TCTR20221109006. Registered 09 November 2022 - Retrospectively registered, https://www.thaiclinicaltrials.org/show/TCTR20221109006.

## Introduction

Teeth with open apices with or without periapical lesions have been treated by many means, from long-term apexification using calcium hydroxide to manipulation of the MTA [1, 2].

Conventional apexification with calcium hydroxide Ca(OH)_2_ was performed long ago and has been recognized as a positive outcome [3]. However, this modality is associated with certain unfavorable conditions, such as prolonged follow-up, multiple visits, and distinctive manipulation [4]. Although this traditional technique is not sensitive, a weakened dentin structure can be an outcome of this uncomplicated and affordable technique. Patient compliance is one of the most important factors for successful use of this modality because reinfection of the canal space results from incorrect follow-up appointments and fracture of the remaining tooth structure [5].

Apical closure using a blood clot and MTA in the root canal system for revascularization or regeneration of the pulp complex is also another promising curative option [6]. However, this procedure has unpredictable outcomes with certain consequences. This revascularization procedure has certain drawbacks when the mass of the dental structure has been previously lost from many causes because the remaining tooth tissue cannot withstand both the para-functional and functional forces.

The prevention of apical extrusion of endodontic substances is challenging for teeth with periapical lesions and wide, open apical foramens. The environment is wet and invisible, and the material is easily extruded beyond the apex because of the large foramina, especially because of the instability and long setting time of the material, such as calcium silicate-based cement and root canal sealer. Although the extrusion of MTA had a nonsignificant effect on the healing of periapical lesions, healing may take a longer time, and this consequence is not supported by clinicians [7].

Thermal or chemical gutta-percha techniques for accessing apical third obturations are ineffective for teeth with periapical lesions and wide, open apical foramens [1].

The formation of the apical barrier of certain biological materials is a prerequisite for predictable, effective manipulation of apical third obturation [1]. Several authors have successfully established that the hemostatic collagen membrane is an effective apical barrier for preventing biological substance extrusion [1, 8].

By introducing platelet-rich fibrin (PRF) [9], especially the new form of PRF, A-PRF+, this strong fibrin membrane enriched with platelets and growth factors can be manipulated commonly in periodontology, implantology, oral surgery, and nonsurgical endodontic therapy [10]. PRF used in root canal therapy focuses on the application for regenerative endodontic procedure, which could not ensure in complete root formation, and modest reduction of root fracture opportunity [11]. PRF is characterized by the relative centrifugal forces (RCFs) and has to being used for proper purposes [12, 13]. Up to now, the investigations in PRF application for teeth with open apices are limited, and focused only on the conventional PRF [10, 14–18]. The PRF showed good biocompatibility and promising results when it was combined with the MTA [6, 11, 15, 17, 19].

The aim of the present pilot study was to evaluate the effectiveness of the combination of MTA and A-PRF^+^ or of MTA alone in the nonsurgical endodontic treatment of teeth with periapical lesions and open apices.

## Methods

The present pilot study was approved by the Research Ethics Committee of the University of Medicine and Pharmacy at Ho Chi Minh City, Viet Nam, with the approval number 236/ĐHYD-HĐĐĐ. The study was also reviewed and approved by the Thai Clinical Trials Registry Committee with the approval number TCTR20221109006. Informed consent was obtained from all participants or their parents, and the method was performed in accordance with the relevant guideline and regulation, following the guideline and regulation of the World Medical Association Declaration of Helsinki: Ethical Principles for Medical Research Involving Human Subjects [20]. Informed consent was also obtained from all patients and/or their legal guardians for the publication of identifying information/figures in an online open-access version.

The sample size was calculated using G*Power version 3.1.9.6 (Universität Kiel, Germany) with an effect size of 0.98, an alpha of 0.1, and a power of 0.8, leading to a sample size of 30 teeth. The Wilcoxon-Mann-Whitney test (two groups) of the family of t test in the software was chosen for the decrease in the dimension of the periapical radiolucent region before and after nonsurgical root canal therapy.

### Sample selection

Tooths on both the maxillary and mandibular sides were collected for the present pilot study. Patients were diagnosed with symptomatic or asymptomatic apical periodontitis, periapical lesions, or open apices.

The subjects were not included in the present pilot study if they had orofacial chronic pain, such as migraine, sinusitis, temporomandibular pain, or trigeminal neuralgia.

Teeth with severe structures that were unrestorable, had unacceptable crown-to-root ratios, or were cracked were also excluded from the present pilot study.

The recruitment of patients was performed at the Department of Operative Dentistry and Endodontics, Faculty of Odonto-Stomatology, University of Medicine and Pharmacy at Ho Chi Minh City, Viet Nam, from November 2021 to May 2023.

Twenty-seven patients with twenty-eight teeth were recruited for the present pilot study. Twenty-eight teeth were randomly distributed into two groups. The PRF group with fourteen teeth was treated with the combination of PRF and MTA Angelus (Angelus, Brazil), and the non-PRF group with fourteen teeth was treated with only the MTA Angelus for an apical barrier.

### Endodontic treatment

All endodontic treatments were performed by the same endodontist via the same standard procedure. For the first appointment, after completing the administrative procedure, a putty silicone bite registration impression was made to capture the first long cone periapical digital radiograph using the X-Ray unit and the phosphor plate (X-Mind, Satelec, Acteon Group, France) with a 16-inch positioning radiography holder (XCP, Rinn, Dentsply Sirona, Switzerland). The ring of the positioning holder was placed at the end of the arm, touching to the end of the cone head of the X-ray unit. Along with the individual putty silicone bite registration impression, the positioning holder ensured the same position of the phosphor plate for every patient in several following periodic examinations.

Local anesthesia was administered using 2% lidocaine (Lignospan standard, Septodont, France), followed by rubber dam isolation (Ash Rubber Dam, Dentsply Sirona, Switzerland). A 3D dental operating microscope (Promise Vision 3D, Seiler, St. Louis, MO, USA) was used for enhanced vision and illumination during the entire root canal procedure.

The endodontic access cavity was prepared using a Martin and Endo-Z bur (Dentsply Sirona, Maillefer, Switzerland) under copious sterile water spray. The root canal length was subsequently measured via the synthesis of information from an electronic apex locator (ProPex PiXi, Dentsply Sirona, Maillefer, Switzerland), a periapical digital radiograph, and observation under a dental operating microscope. The working length was shorter than the root canal length at 2 mm. The root canal was prepared using K-files (Dentsply Sirona, Maillefer, Switzerland) with gentle, circumferential motion. An irrigation needle (Max-i-Probe, Dentsply Sirona, Maillefer, Switzerland) was used to deliver 3% sodium hypochlorite (Canal Pro, Coltene Whaledent, Altstätten, Switzerland) into the root canal space for copious irrigation.

Root canal preparation was performed with the purpose of at least dentin removal and as much content cleaning. Saline solution was used for final irrigation of the root canal. After being parched by sterile paper points, the root canal was filled with calcium hydroxide paste (Endo Cal, Septodont, France), which was subsequently gently condensed using a proper plugger (Dentsply Sirona, Maillefer, Switzerland), ensuring that the paste fully occupied the entire root canal, from the apical end to the cemento-enamel junction. A temporary material (Cavit, GC, Tokyo, Japan) was used to fill the access cavity with an underlying sterile cotton pellet.

The next appointment was scheduled for one week after the first visit. The apical plug procedure was prepared after the completion of the root canal content removal and drying.

For the PRF group, ten milliliters of venous blood were drawn from the subject’s vena brachia. The fibrin clot containing the platelets was extracted from the patient’s blood using an apparatus at 1300 rpm for 8 min, with RCF_clot_ of 145 [12, 13] (PRF Duo Quattro, Nice, France). The PRF membrane was created using the cover of the box with no further pressure on the cover in 120 s.

The PRF membrane was cut into pieces approximately 3 mm × 3 mm in length, inserted, and gently condensed into the apical apex region through the apical foramen with an appropriate plugger. Under the dental operating microscope, the pieces of cut PRF were condensed incrementally through the apical foramen into the periapical area using the proper condenser until a firm barrier was established at the apex of the root. The procedure was performed rapidly and easily because of the widening of the apical portion of the root. The position and texture of the PRF barrier were checked for stability with a plugger under a dental operating microscope, and additional pieces of PRF could be further condensed into place if needed.

The MTA HP Repair Angelus (Angelus, Brazil) was mixed following the manufacturer’s instructions and inserted into the MTA carrier by digging the gun into the prepared MTA material mass. The MTA material was delivered into the apical region by triggering the piston of the carrier. The plugger was used to gently pack the material against the PRF barrier until 5 mm of MTA was placed. The placement of MTA was checked for appropriateness or any material extrusion by digital radiography.

The remaining canal space was obturated with thermoplastic gutta-percha (EQV, Meta Biomed, Korea) and AH Plus sealer (Dentsply Sirona, Maillefer, Switzerland). A composite and bonding system (GC, Tokyo, Japan) was used to restore the access cavity after ensuring obturation quality via digital radiography.

For the non-PRF group, nonsurgical endodontic treatment was performed as described for the PRF group, but the pieces of the PRF membrane were not used for apical matrix formation.

### Periodic follow-up examination

The periodic follow-up periods of 1, 3, 6, and 9 months were scheduled for clinical and radiographical examinations (using the individual bite registration impressions). Clinical symptoms such as postoperative pain and analgesic consumption, pain upon palpation or percussion, and sinus tract were recorded. Treatment failure was confirmed if any of the above clinical symptoms existed. Certain dimensions of the periapical lesion were measured to obtain the data for analysis. The dimension was determined as the largest distance of the segment from the two intersections between the lesion circumferential and the line drawn perpendicular to the root axis. Radiographic success was confirmed if the dimension was reduced or unchanged. All measurements were conducted by the same operator, and the kappa (for clinical symptoms) or intraclass correlation (for radiographic dimensions) coefficient was used to evaluate intraexaminer agreement.

All the statistical analyzes were performed using IBM SPSS Statistical Software version 27 (IBM, Armonk, NY, US), with a significance level of *p* < 0.05. The data were first tested for normality by the Shapiro–Wilk test. If the data were not normally distributed, they were analyzed using the Friedman and Dunn–Bonferroni post hoc correction.

## Results

The demographic data, categories and diagnosis of the teeth in the sample are displayed and analyzed in Table 1.

**Table 1:** The demographic data, categories, and diagnosis of the teeth in the experimental sample

Group		PRF	Non-PRF	*P*
Age		14 ± 5.4 (yrs)	21.3 ± 7.9 (yrs)	0,006*
Gender				
	Male	7	10	0.462
	Female	8	5	
Categories				
	Incisor	3	9	0.060
	Premolar	12	6	
Cause				
	Injury	3	7	0.245
	Occlusal supernumerary cusp	12	8	

Yrs: years

*P < 0.05, Fisher exact test

There was a significant difference in the age of patient between the two experimental groups.

The widths of the periapical lesions in the two experimental groups are displayed and analyzed in Table 2.

**Table 2:** The horizontal dimensions of the periapical lesions in the two experimental groups

	Minimum	25th percentile	Median	75th percentile	Maximum	Friedman test
T_0w_	3,50	4,6400	6,0500	7,2200	9,87	< 0,0001*
T_1w_	0,67	3,2600	3,5000	4,3800	5,22	
T_3w_	0,00	0,0000	0,0000	1,2300	3,87	
T_6w_	0,00	0,0000	0,0000	0,0000	3,39	
T_9w_	0,00	0,0000	0,0000	0,0000	2,60	
T_0wo_	2,41	2,9800	5,6900	7,5200	12,08	0,0001*
T_1wo_	1,46	2,5000	4,7200	7,3900	8,38	
T_3wo_	0,00	0,0000	3,8900	7,3800	10,20	
T_6wo_	0,00	0,0000	1,9500	6,5900	9,67	
T_9wo_	0,00	0,0000	1,2800	5,2500	8,76	

*T*_*0w*_, *T*_*1w*_, *T*_*3w*_, *T*_*6w*,_*and T*_*9w*_: *The periapical lesion width of the PRF group at the beginning, first, third, sixth, and the ninth month, respectively*

*T*_*0wo*_, *T*_*1wo*_, *T*_*3wo*_, *T*_*6wo*,_*and T*_*9wo*_: *The periapical lesion width of the non-PRF group at the beginning, first, third, sixth, and the ninth month, respectively*

* *P* < 0.05, Friedman test

There were significant differences in the width of the periapical lesion on digital radiography between the experimental groups at different time points.

The pairwise comparisons with Bonferroni correction for the width of the lesions on the digital radiographs are displayed in Table 3.

**Table 3:** Pairwise comparisons of the width of the periapical lesions on the radiographs obtained through periodic examinations

	Test Statistic	Standard Test Statistic	Significance	Adjusted Significance^⁑^
T_9w_ - T_6w_	0,133	0,231	0,817	1,000
T_9w_ - T_3w_	0,767	1,328	0,184	1,000
T_9w_ - T_1w_	2,300	3,984	0,000	0,001*
T_9w_ - T_0w_	3,300	5,716	0,000	0,000*
T_6w_ - T_3w_	0,633	1,097	0,273	1,000
T_6w_ - T_1w_	2,167	3,753	0,000	0,002*
T_6w_ - T_0w_	3,167	5,485	0,000	0,000*
T_3w_ - T_1w_	1,533	2,656	0,008	0,079
T_3w_ - T_0w_	2,533	4,388	0,000	0,000*
T_1w_ - T_0w_	1,000	1,732	0,083	0,833
T_9wo_ - T_6wo_	0,200	0,346	0,729	1,000
T_9wo_ - T_3wo_	0,700	1,212	0,225	1,000
T_9wo_ - T_1wo_	1,767	3,060	0,002	0,022*
T_9wo_ - T_0wo_	2,500	4,330	0,000	0,000*
T_6wo_ - T_3wo_	0,500	0,866	0,386	1,000
T_6wo_ - T_1wo_	1,567	2,714	0,007	0,067
T_6wo_ - T_0wo_	2,300	3,984	0,000	0,001*
T_3wo_ - T_1wo_	1,067	1,848	0,065	0,647
T_3wo_ - T_0wo_	1,800	3,118	0,002	0,018*
T_1wo_ - T_0wo_	0,733	1,270	0,204	1,000

*T*_*0w*_, *T*_*1w*_, *T*_*3w*_, *T*_*6w*,_*and T*_*9w*_: *The periapical lesion width of the PRF group at the beginning, first, third, sixth, and the ninth month, respectively*

*T*_*0wo*_, *T*_*1wo*_, *T*_*3wo*_, *T*_*6wo*,_*and T*_*9wo*_: *The periapical lesion width of the non-PRF group at the beginning, first, third, sixth, and the ninth month, respectively*

^⁑^ *Significance values were adjusted by the Bonferroni correction for multiple tests*

* *P* < 0.05, Dunn-Bonferroni post hoc correction

There were significant differences in lesion width between the diagnostic examination and the third month examination in both the PRF group and the non-PRF group (*P* < 0.05).

The lesion width differences between the two experimental groups are displayed and analyzed in Table 4.

**Table 4:** The width differences between the two successive periodic examinations of the two experimental groups

	Mann-Whitney U	Wilcoxon W	Z	Exact Significance
T_0w_ - T_0wo_	103,000	223,000	-0,394	0,713
T_1w_ - T_1wo_	93,000	213,000	-0,809	0,436
T_3w_ - T_3wo_	69,000	189,000	-1,903	0,074
T_6w_ - T_6wo_	60,500	180,500	-2,570	0,029*
T_9w_ - T_9wo_	58,000	178,000	-2,788	0,023*

*T*_*0w*_, *T*_*1w*_, *T*_*3w*_, *T*_*6w*,_*and T*_*9w*_: *The periapical lesion width of the PRF group at the beginning, first, third, sixth, and the ninth month, respectively*

*T*_*0wo*_, *T*_*1wo*_, *T*_*3wo*_, *T*_*6wo*,_*and T*_*9wo*_: *The periapical lesion width of the non-PRF group at the beginning, first, third, sixth, and the ninth month, respectively*

* *P* < 0.05, Mann-Whitney U test

There were significant differences between the experimental groups in the sixth month in terms of the width of the periapical lesion on digital radiographs.

There were seven teeth with periodontal ligament widening at the first examination and there was no tooth with this symptom in the third month of the periodic examination in the PRF group. There were nine teeth with widened periodontal ligament at the first examination and there were still five teeth with widened periodontal ligament in the ninth month of periodic examination in the non-PRF group.

## Discussion

The results of the present pilot study showed that all treated patients healed at different levels at periodic evaluations of both periapical digital radiographs and clinical signs. The periapical lesion was largely cured in the third month of the both groups. Lesion width was smaller in the PRF group than that in the non-PRF group, and there were significant differences in lesion width between the PRF group and the non-PRF group at the sixth and the ninth months.

A dental operating microscope with proper magnification is essential for every endodontic procedure, especially for this complicated procedure. Indirect vision in the dark, wet, complicated apical region requires a powerful light source and suitable magnification that cannot be achieved by conventional dental light or a normal head loupe even with enhanced illumination [21, 22]. With the advancements in 3D dental operating microscopes used in the present pilot study, motion pictures of the field of operation were displayed on a 3D monitor accompanied by 3D glass. The position of the 3D monitor could have been easily mechanically adjusted by pressing a button to obtain the most comfortable posture for the clinician [21]. This modality enhances the working conditions of operators and helps surgeons become more comfortable and improves dental nonsurgical endodontic education. In addition, the body of the main microscope could have been rotated or displaced easily and freely to obtain the best images from the field of operation. Regardless of the position of the main body of the microscope, the posture of the clinician could not change because of the stable 3D monitoring position [21]. This approach helps patients obtain more comfortable postures during long endodontic procedures. In addition to one HDMI port for a normal video signal, there is another HDMI port for a 3D video signal on a 3D dental operating microscope for the 3D option in dental education for both students and patients. The most important advantage of this special modality is the flexibility of the operator at every magnification, even at the highest magnification. The operator could easily, quickly and comfortably return to the working area whenever he or she needs to after certain intermittent movements. The clinician could have their eyes focused on every target but working area, regardless of the reason. The operator could easily and quickly grasp any small instruments without help from the assistant.

One disadvantage of the 3D dental operating microscopes is the ability to manipulate light transmission optic fibers [21]. However, the light intensity in the working area has been sufficient for every duty in the endodontic procedure. The durability of the camera system in 3D dental operating microscopes in special high-humidity environments such as dentistry has been questionable and needs to be confirmed by the manufacturer, although there are no issues during the manipulation of 3D microscopes in the present pilot study. High cost, difficult maintenance and unavailability of the present 3D dental operating microscope have also been other disadvantages of this modality.

The light intensity from the 3D dental operating microscope has been considered because of the periapical living tissue in the working area. There are many ways to adjust the light intensity in the field of view [21–24]. The simplest approach has been to adjust the mirror to direct the light beam in another direction. Intermittent illumination of the working area by turning the light source on or off has been another option for reducing the amount of light energy absorbed by living tissue. The reduction of the light intensity by the adjusted knob on the body of the microscope has also been a proper choice. Manipulation of the contrast filter on the microscope has also been a wise option for light energy reduction [22, 23].

The fear of blood sample collection has been an important factor preventing the enrollment of the subjects [25]. In Vietnamese culture, preparing blood samples for examination purposes has been considered unacceptable. Using ten millimeters of blood for PRF extraction was also another barrier to recruiting the subjects for the present pilot study [25]. For these reasons, the present pilot study has been expanded longer than expected.

The PRF membrane with RCF_clot_ of 145 used in the present pilot study was constructed with a box instrument accompanied by a centrifuge machine [26]. Steel plane of the box cover and the basement proper designate the PRF membrane once it is withdrawn from the sterile vacuum tube. The dimension of the piece of the PRF membrane correlates with the size of the apical foramen for each circumstance and facilitates the manipulation of the insertion through the foramen [26]. The size of the plugger is suitable for the apical foramen because it does not damage the delicate dentinal wall of the apical third further. Patient posture during the procedure is certainly uncomfortable because of the time-consuming nature of the procedure and the unfavorable mandibular tooth position [22].

The results of the present pilot study revealed that although PRF is an autogenous substance, the healing process time is not rapid enough compared with that of the collagen membrane used in a previous study [1]. PRF promotes successfully tissue regeneration and healing process because of its platelets rich in growth factors and cell migration ability [19]. Although PRF has certain advantages over the collagen membranes, the manipulation of this autogenous material requires a strictly sterilized environment, leading to difficulty in normalizing the procedure in common clinical situations [12, 13, 19].

The MTA used for the apical plug is chosen for discoloration of the dental structure, especially for the anterior tooth [1]. Although calcium silicate-based cement is placed only in the apical third, discoloration of the coronal portion of the tooth should be considered an adverse effect during this procedure [27]. Modern MTA erases the discoloration of teeth and is a reasonable reason for its use in the clinical setting. The moisture environment required for the setting of the conventional MTA is eliminated in the manipulation of this modern MTA; therefore, the following appointment for treatment completion is unnecessary [27]. Water is rapidly separated from the PRF membrane; therefore, this substance should be manipulated as soon as possible to establish a parched region, facilitating subsequent MTA placement [17]. The volume of the PRF barrier should be extended further into the apical region to compensate for the shrinkage of the PRF block after compression by sterile cotton.

Thermo-plasticized gutta percha technique is not recommended for the tooth with periapical lesion and open apex, without apical barrier matrix because of wide apical foramen and uncontrolled length of working distance [1, 10, 16, 17, 28]. Once the apical plug of MTA has been established, any obturation techniques could have been performed to fill the remaining space of the root canal. Thermo-plasticized gutta percha technique has been chosen because of its non-invasive mechanism of the technique and high-speed manner [28]. This obturation technique is appropriate and acceptable when the post space is needed for both indirect or direct restorative procedure [28].

Because of the uniqueness of the present nonsurgical endodontic technique, PRF extraction has been performed as a sensitive procedure to ensure timeliness of the whole procedure. The PRF block and pieces of the PRF membrane were immediately and freshly manipulated for insertion into the periapical region [17].

The apical MTA barrier combined with the PRF membrane offers a high chance of successful restoration of extremely weakening tooth structure when compared with other long-term, multiple visits, such as calcium hydroxide apexification and apical closure regeneration [3]. The texture of PRF membranes seems tougher than that of collagen membranes under the conditions of these PRF extraction parameters [1]. This characteristic facilitates the manipulation of PRF pieces in the delicate apical region, although water separates from the compressed substance during placement.

The estimation of the blood volume needed for PRF extraction is not an issue if a previous cone-beam computed tomography image is available. The dimensions of the periapical lesion could be estimated more exactly via CBCT than via conventional periapical digital radiography, as described in the present pilot study. However, radiation exposure is an issue whenever CBCT is used, preventing further manipulation of this measure in the present pilot study [29]. CBCT has also been a useful and invaluable modality for evaluating treatment outcomes because of its 3D-constructed characteristics. However, CBCT has not been considered a routine imaging method, especially in endodontics, because of the low likelihood of possible elimination (ALARA) principle [30]. Patient safety was required for all the above factors, regardless of any benefit, even for the patient. Provided that CBCT safety has not been safely established, the routine use of CBCT in endodontics has not been delayed [29].

The reduction in the lesion horizontal dimension in the PRF group was greater than that in the non-PRF group in the present pilot study. The reason could be the favorable environment of the periapical region in the PRF group, which included growth factors, antibacterial agents and the apical barrier preventing the extrusion of MTA over the apex.

There was further elongation of the apices in the PRF group, as described in a previous study [3]. Thickened canal walls were observed in certain patients in the PRF group [3]. There were certain differences in the ages of the subjects in both experimental groups. However, these results support the concept of manipulating PRF as an apical barrier in combination with placing MTA.

PRF membrane formation was confirmed using the cover of the accompanying box in the kit without further requirement. A PRF piece cut from the PRF membrane from the large membrane facilitates the insertion of the piece over the large apical foramen under a dental microscope [17, 18, 26]. The technique is sufficiently tough and small for manipulating material in the complicated periapical area. Manipulating the MTA with an apical lesion is challenging. Although the operator can have excellent vision, magnification, and illumination under a dental microscope, preventing the extrusion of MTA is difficult [17, 18, 26].

The utilization of the hemostatic collagen membrane, as described in previous studies, is convenient, fast, and less invasive because of the availability, ease of manipulation, and low cost. This dedicated approach means success in certain circumstances [1]. However, the results seem modest when compared with those of the present pilot study. The healing of the lesions was slower, and further formation of the apex was fainter than that in the present pilot study.

Both MTA and PRF are popular in dentistry. However, MTA is very popular, while PRF is less popular than the former in endodontics [10, 17, 18]. PRF is encountered mostly in surgical therapy, such as oral surgery, periodontal open flap management, and implant procedures [13, 26].

The piece of PRF membrane for an apical matrix in the present pilot study can fulfill the requirements of excellent autografts for both mechanical and biological purposes [17, 18]. Once the PRF apical barrier has been firmly established, the placement of MTA afterward becomes easier than ever [17, 18]. MTA could have been easily and quickly inserted into the apical third region by any means without material extrusion. The operator could have used the MTA carrier, hand-use, or even an engine-driven spiral instrument. The condensation of MTA after placing it into the apical third region could have been performed by a proper hand-use plugger or a WaveOne Gold paper cone of enormous size. Other authors suggest the condensation of MTA by using an ultrasonic condensation instrument after a better result is achieved, although there was no significant difference between manual and ultrasonic condensation [31]. The discoloration of the aesthetic tooth structure is not considered with respect to the new MTA materials, as is currently the case for Angelus MTA. With improvements in the composition of the material, the new MTA could be used for teeth in both the posterior and anterior Sect [27].

After several years of endodontic treatment for periapical lesions with hydroxide calcium, the healing process is normally elongated and unpredictable. Manipulation of the combination of MTA with PRF is a promising means to reduce the treatment process and healing period [27].

One of the most important advantages of the contemporary procedure used in the present pilot study is the ability to reconstruct previously missing tooth structures as soon as possible immediately after the last step of the procedure. This ensures the important aesthetic and functional duties of the concerned tooth [3]. The present pilot study is one of the first investigations in using the combination of A-PRF^+^ and MTA for treatment of teeth with open apices [14–18].

The PRF is an autogenous, safe, inexpensive and accessible substance in every patient for such a procedure described in the present pilot study. Manipulation of the PRF is not so complicated and easy to learn for the clinician with the dental operating microscope, an indispensable device of the endodontist. With the formation of the external apical matrix, the MTA would be ensured to be confined inside the root canal, preventing the irritation of the bioceramic material to the periapical region because of the extrusion of the material. The acceptable results of the present pilot study promote the manipulation of the combination between PRF and the MTA in the treatment of teeth with periapical lesions and open apices.

The limitations of the present pilot study include the small sample size, short follow-up period, small number of teeth, and the use of only periapical digital radiographs. Further investigations should be performed with the larger sample size and longer follow-up period, using other calcium silicate-based materials, other PRF forms, and a broader range of teeth and ages.

The effect of fluid separation from the PRF block on membrane formation was further investigated to confirm the effectiveness of the PRF membrane in this way whenever needed for procedures.

Further investigations of other biological materials and other PRF forms have been performed in older subjects to obtain deeper and clearer knowledge of these biological materials in nonsurgical endodontic therapy.

The PRF membrane is a promising autogenous substance for the apical barrier formation procedure for preventing apical extrusion of material used in the apical region. Small number of study subjects and the short time of follow-up period limit the generalizability of these results.

## Data Availability

The data that supports the findings of this study are available from the corresponding author upon reasonable request.

## Abbreviations

3D: Three Dimension
ALARA: As Low As Reasonably Achievable
HDMI: High-Definition Multimedia Interface
MTA: Mineral Trioxide Aggregate
PRF: Platelet Rich Fibrin
RCF: Relative Centrifugal Forces
